# Complete mitochondrial genome of the drywood termite *Cryptotermes havilandi* (Isoptera: Kalotermitidae)

**DOI:** 10.1080/23802359.2021.1873710

**Published:** 2021-02-11

**Authors:** Petr Stiblik, Pierre Dieudonné Akama, Jan Šobotník

**Affiliations:** a Faculty of Forestry and Wood Sciences, Czech University of Life Sciences Prague, Prague, Czech Republic; b Département des sciences biologiques, Ecole normale supérieure, Université de Yaoundé I, Yaoundé, Cameroon; c Faculty of Tropical AgriSciences, Czech University of Life Sciences Prague, Prague, Czech Republic

**Keywords:** Kalotermitidae; termite mitochondrial genome; isoptera; drywood termites

## Abstract

We report the first complete mitochondrial genome of an important pest of timber, the drywood termite *Cryptotermes havilandi*. The gene content and synteny of the mitochondrial genome of *C. havilandi* is identical to that of other termite species reported to date. It is composed 13 protein-coding genes, two ribosomal RNA genes, and 22 transfer RNA genes. Our phylogenetic tree, that includes the mitochondrial genomes of 14 species of Kalotermitidae, including *C. havilandi*, resolves the phylogenetic position of *C. havilandi* within Kalotermitidae.

## Main text

*Cryptotermes havilandi* Sjöstedt, 1900 (Isoptera: Kalotermitidae) is an important pest of structural lumber and sheltered wood (Su and Scheffrahn 2000). Although it is now distributed across the tropical and subtropical regions, *C. havilandi* originated from Africa, and has been introduced outside its native range largely by the intermediary of human transportation (Evans 2011; Evans et al. 2013). It is now invasive in various Caribbean islands, Guiana, Surinam, Brazil, Madagascar, the Comores, and India (Evans et al. 2013). Despite its economic importance, the mitochondrial genome of *C. havilandi* has not been sequenced yet. Here, we provide the first complete mitochondrial genome sequence of a *C. havilandi* extracted from the sample CAM101 collected on 7th of April 2015 in an abandoned wooden house in northern Cameroon, Africa (N04°42′25″ E009°43′08″), by the authors.

We sequenced *C. havilandi* (GenBank: MW208858) mitochondrial genome using Illumina HiSeq2000. The genome was assembled using the clc suite of programs as described by Bourguignon et al. (2015). The total length of the complete mitochondrial genome of *C. havilandi* is 15,559bp. As in other mitochondrial genomes of termites previously sequenced (Cameron and Whiting 2007; Cameron et al. 2012; Bourguignon et al. 2015, 2016, 2017; Wu et al. 2018; Wang et al. 2019), the mitochondrial genome of *C. havilandi* is composed of 13 protein-coding genes (following the order: *nad2, cox1, cox2, atp8, atp6, cox3, nad3, nad5, nad4, nad4l, nad6, cytb,* and *nad1*), two ribosomal RNA genes (*rnl* and *rns*) and 22 transfer RNA genes (following the order: *Ile, Gln, Met, Trp, Cys, Tyr, Leu^(UUR)^, Lys, Asp, Gly, Ala, Arg, Asn, Ser^(AGN)^, Glu, Phe, His, Thr, Pro, Ser^(UCN)^, Leu^(CUN)^,* and *Val*). The GC-content is 34%. Our results confirm that termite mitochondrial genomes are stable in gene content and preserved their synteny.

**Figure 1.**
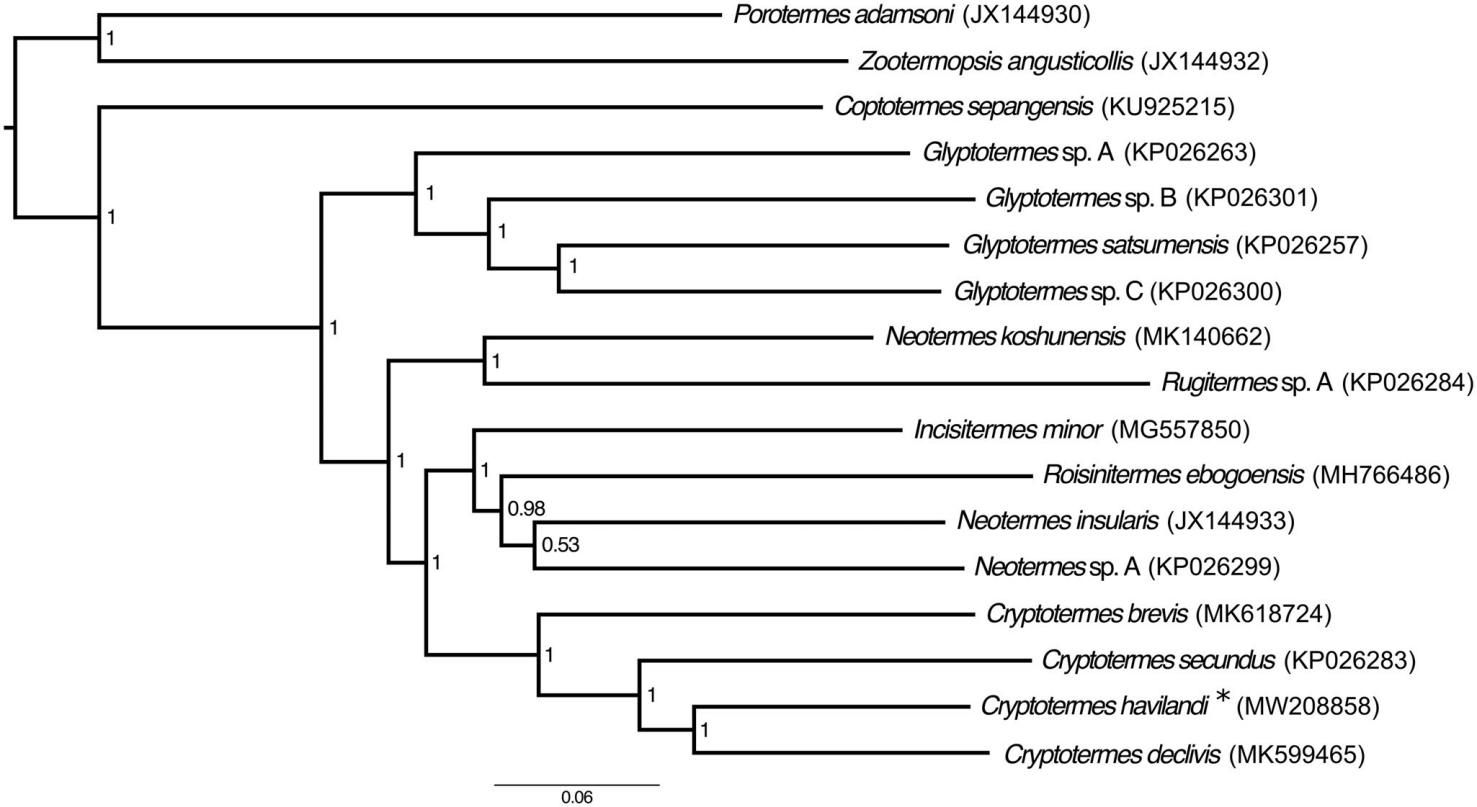
Bayesian phylogenetic tree of all species of Kalotermitidae sequenced to date. Numbers in nodes state for posterior probabilities and the scale indicates 6% genetic variation for its length. GenBank accession numbers are given in brackets. “*” mark the studied species.

To shed light on the phylogenetic position of *C. havilandi* within the Kalotermitidae, we reconstructed a phylogenetic tree that included all mitochondrial genomes of Kalotermitidae sequenced to date, including the mitochondrial genome of *C. havilandi*, and three outgroups: *Zootermopsis angusticolis* (Isoptera: Archotermopsidae)*, Porotermes adamsoni* (Isoptera: Termopsidae) and *Coptotermes sepangensis* (Isoptera: Rhinotermitidae) (Figure 1). All genes were aligned separately using MAFFT v. 7.3 (Katoh and Standley 2013), concatenated, and the phylogenetic tree was reconstructed using MrBayes v. 3.2.1 (Ronquist et al. 2012). The parameters of the phylogenetic analysis were set as described by Bourguignon et al. (2017). Overall, our phylogenetic tree confirms the monophyly of *Cryptotermes*, within which *C. havilandi* is nested.

The genus *Cryptotermes* includes several invasive species that cause major economic losses in the world (Evans et al. 2013). Surprisingly, very few studies have used molecular markers to study the population genetics of *Cryptotermes* species. In this paper, we provide the mitochondrial genome of one of the most important termite pest. The new mitochondrial genome presented here will help to understand how the major termite pests have been introduced around the world.

## Data Availability

The genome sequence data that support the findings of this study are openly available in GenBank of NCBI at (https://www.ncbi.nlm.nih.gov/) under the accession no. MW208858. The associated SRA, BioProject, and Bio-Sample numbers are SRR13287752, PRJNA687161 and SAMN17119085, respectively. The sample CAM101 is available in termite collection at Czech University of Life Sciences, Prague, Czech Republic in both states, as an 80% ethanol voucher sample and in RNAlater preservative (contact: stiblik@fld.czu.cz).
